# The Pathology of Milk-Sickness, in Parke County, Ind.

**Published:** 1857-06

**Authors:** John Pickard


ARTICLE III.—*The Pathology of Milk Sickness, in Parke County, Ind.* By Dr. JOHN PICKARD.

Thinking the pathology of milk sickness, as it appears in Parke County, Ind., taken from observations of citizens who have been acquainted with it for thirty years, might be interesting to some of the readers of the *Journal*, I have prepared the following imperfect article. So little is known by our book making physicians that they give us no information worthy of note; and so contradictory are the investigations of physicians, who have come in contact with it, that it is impossible to come to any definite conclusions, from the reports we find in the journals. The views given in the April No. of the *Journal*, relating to the Etiology of the disease under consideration, very nearly correspond with the following experiments, which conclusively show that it is not vegetable, animal, or malarial. A farmer living about a mile from where the disease is known to exist, allowed his cattle to run out, but they never reached the infested district until the dew evaporated from the vegetation; the result was, his cattle nor his family were ever attacked by the disease; were it a vegetable, the absence of the dew would not destroy its poisonous qualities.

Another family having suffered from its ravages, plowed up a pasture field, digged around the stumps, thoroughly turning all the soil, and sowed the field in grass, upon which they have kept their stock for twenty years, and at no time has milk sickness made its appearance; while on other portions of the farm uncultivated, it is as fatal as ever. Whether the poison is a mineral or a gas is yet to be tested; but I am inclined to believe it a gas much heavier than the atmosphere, consequently resting upon the vegetation; dry weather seems to favor its production. Last season it was prevalent in many districts, where it had not made its appearance for a number of years, but in no instance did it attack animals kept upon cultivated ground. It may be in the water, but we are not acquainted with any district where this has been positively proven. It, however, seems improbable when the disease prevails upon the neplands, and the springs from which the stock drink flow through the bottom lands, and is used there by other stock, yet the disease never makes its appearance among them.

All species of animals are liable to be attacked by milk sickness, the gramnivera, first, and among those the sheep appears to be the most easily affected; the carnivera always obtain it from the flesh of animals that have died with the disease. It is often concentrated in the milk of the cow, and the calf will be attacked when the cow shows no symptoms of disease. Animals well fatted are not liable to be attacked. A farmer in this vicinity, kept his horses on a lot of one acre, and fed them on hay and oats, the disease soon made its appearance among them; he then turned his hogs upon the same lot, fed them all the corn they would eat, and they showed no symptoms of the malady. From the observations and experiments which have been made where the citizens have suffered severely from the loss of property, and the lives of those still dearer to them, we deduce the following conclusions. 1st. Milk sickness is caused by a poison, this poison is generated near the surface of the earth, from some peculiarity in the soil or the soil and atmosphere combined. 2d. That the poison is a mineral or heavy gas, which appears to be produced in the night and destroyed by the rays of the sun. 3d. A thorough cultivation of the soil will destroy it. 4th. That it may be communicated from one animal to another by the flesh or milk. And 5th, that oleaginous substances act as a preventive, and will often cure animals affected with the disease. There is no difficulty in the diagnosis, the patient invariably complains of great weakness, inability to perform labor, a small amount of exercise produces trembling and fainting sensations; as the disease advances, vomiting, and obstinate costiveness are invariable accompaniments, and during the whole time a peculiar odor pervades the room, unlike anything we have ever met with.

The treatment of milk sickness is simple; we have previously stated, that oleaginous substances are preventives, and we have found no remedy so efficient as Aleum Ricini, by the stomach, if possible, if not give enemata. Apply sinapisms to the stomach, give cooling mucilaginous drinks, overcome the costiveness as soon as the nature of the case will admit, and you have conquered the main obstacle to successful cure.

